# Comparison of responsiveness to cancer development and anti-cancer drug in three different C57BL/6N stocks

**DOI:** 10.1186/s42826-019-0015-z

**Published:** 2019-10-04

**Authors:** Mi Ju Kang, Ji Eun Kim, Ji Won Park, Hyeon Jun Choi, Su Ji Bae, Kil Soo Kim, Young-Suk Jung, Joon-Yong Cho, Dae Youn Hwang, Hyun Keun Song

**Affiliations:** 1 Department of Biomaterials Science, College of Natural Resources & Life Science/Life and Industry Convergence Research Institute, Pusan National University, 50 Cheonghak-ri, Samnangjin-eup Miryang-si, Gyeongsangnam-do 627-706, Miryang, 50463 Korea; 2 College of Veterinary Medicine, Kyungpook National University, Daegu, Korea; 3 College of Pharmacy, Pusan National University, Busan, Korea; 4 Exercise Biochemistry Laboratory, Korea National Sport University, Seoul, Korea; 5 Central Research Institute, Kine siences Co., F1, Milovany, Goryeodae-ro 28, Seongbuk-gu, Seoul, Korea

**Keywords:** C57BL/6N, C57BL/6NKorl, Cisplatin, Syngeneic tumor model

## Abstract

In our efforts to understand the systemic features of tumors, the importance of animal models is increasing due to the recent growth in the development of immunotherapy and targeted therapies. This has resulted in increased attention towards tumor animal models using C57BL/6N, which are mainly used in immunological studies. In this study, the C57BL/6NKorl stock and two other commercial stocks (C57BL/6NA and C57BL/N6B) are evaluated by comparing the occurrence of tumors using the syngeneic model; furthermore, we compare the response to anti-cancer drugs in the syngeneic model by evaluating survival, growth of tumors, proliferation and molecular biology analysis. In the syngeneic model using LLC (Lewis lung carcinoma) cells, the survival of mice and growth of the tumor showed a better response in the C57BL/6NKorl stock, and was dependent on the cell concentration of the dosing tumor, as compared to the other C57BL/6N stocks. However, the Ki-67 staining showed only little difference in cell proliferation within the tumor tissue each mouse stocks. Comparing the sensitivity to anti-cancer drug by examining changes in growth, volume and weight revealed that cisplatin treatment for tumor-bearing C57BL/6NKorl was more dependent on concentration. The Ki-67 staining, however, showed no difference among the C57BL/6N stocks after cisplatin treatment. The expressions of p27 and p53 tumor suppressor proteins, caspase-3 and Bax showed dose-dependent increase after exposure to cisplatin, whereas the expression of Bcl-2 was reduced in a dose-dependent manner. Furthermore, the expressions of MMP-2 and VEGF involved in metastasis, as well as inflammatory genes IL-1β, IL-6 and IL-10, showed dose-dependent decrease in tumor tissue after cisplatin exposure. Differences observed among the C57BL/6N stocks were not significant. Taken together, our studies reveal that C57BL/6NKorl has the potential of being a useful biological resource established in Korea, as it does not differ from the two commercially available C57BL/6N stocks when considering response to tumor generation and sensitivity to anti-cancer drugs using the syngeneic tumor model.

**Electronic supplementary material:**

The online version of this article (10.1186/s42826-019-0015-z) contains supplementary material, which is available to authorized users.

## Introduction

The global increase in cancer incidences is attributed to a rapidly aging population and numerous causes, including environmental pollutants. In Korea, cancer incidence and death rates have increased since 2002 due to advanced aging in the society, with social and economic losses ranging from 2000 to 2010 reaching $20,858 million [1, 2]. Numerous researches have been conducted for cancer therapy; from basic research to clinical intermediation studies including surgery, chemotherapy and radiotherapy, and recent studies on immunological therapy such as target therapy and immune checkpoint inhibitors. The diversification of cancer treatments and immunotherapy, in which immunological concepts have been introduced, increases the need for animal models for immunological understanding as well as research in the biology and genetics of human cancer. Many studies are therefore focusing on the development of efficient and useful animal models.

In general, animal model research is widely used in investigating the therapeutic efficacy and toxicity of chemicals and biological materials. Accumulated findings provide insights into the genetic mechanisms of cancer treatment and cancer prevention, malignant transformation and cancer progression. The goal of the cancer animal model is to closely reconstruct human malignancy, and apply it as a systemic tool with a greater likelihood of successfully translating the basic knowledge of the treatment in prevention of cancers in humans. The mouse has been a traditional animal model for basic and systemic cancer research, and is divided into genetically engineered models, syngeneic models and xenogeneic models [3–7]. Genetically engineered models include mice with certain genes missing or inserted through techniques such as transgenic transformation. In the xenograft mouse model, a different species of tumor is transplanted into a mouse in which the immune response is suppressed. In this model, there are difficulties in accurate immunological research because the mouse is immune suppression. The syngeneic mouse tumor model is generated by transplanting isolated tumors from the same origin mice. Under experimental conditions, such as exposure to various anti-cancer drugs, the survival of the mouse, tumor size, toxicity, and molecular biological analysis are the basic parameters studied. The immune response of the mouse is visible as a tumor in the normal state that has not been removed, and has features such as a tumor microenvironment, which is useful for immunological understanding and interpretation. Therefore, recent studies of immunotherapeutic anti-cancer drugs have generated attention as a crucial mechanism [8].

The C57BL/6 mouse is an inbred strain derived from the C57BL mouse, along with the lower mouse strain C57BL/10. Important line separation occurred in 1957 between C57BL/6 and C57BL/10. The well-known C57BL/6J and C57BL/6N were developed from C57BL/6 at the Jackson Institute and the National Institutes of Health in the 1940s and 1950s, respectively [3, 8]. Since C57BL/6 mice have been used from the beginning of researches to establish mice strains for anti-cancer and immunological studies [9], the basic immunological properties of the C57BL/6 mouse are well known. For example, the activity of NK cells is relatively high, as is the activation of helper T cells (such as TH1) which secrete cytokines that promote cell-mediated immunity. It has been reported that the humoral immunity of C57BL/6 shows a lower incidence than that of the Balb/c mice [10–15]. Based on these well-known immunological properties, various cells of C57BL/6 origin, such as EL4, B16F10 and Lewis Lung Carcinoma (LLC), are applied to the syngeneic mouse model of C57BL/6. In this study, we used a syngeneic mouse model of C57BL/6N to evaluate different stocks of C57BL/6N using LLC, a well-established cancer cell line that has the characteristics of metastasis but does not transmit to the lungs subsequent to muscle injection.

Cisplatin (Cis-diamine dichloroplatinum (II), Pt (NH_3_)_2_Cl_2_), which exerts its anti-cancer effect by inhibiting cell division, was used in the treatment of various solid cancers including breast cancer, ovarian cancer, bladder cancer, stomach cancer and lung cancer [16]. A syngeneic mouse model was established in each C57BL/6N stock using the LLC cancer cell line, and the response of each stock-specific anti-cancer was evaluated using cisplatin. Our research provides enough evidence that the C57BL/6NKorl stock and two commercial stocks (C57BL/6NA and C57BL/N6B) have similar characteristics with respect to the occurrence of tumors by syngeneic models and their responses to anti-cancer agents, and they have sufficient competitiveness as the syngeneic model of C57BL/6NKorl stock.

## Materials and methods

### Cell culture

LLC cell line was cultured in Dulbecco’s modified eagle medium (DMEM, GIBCO, Invitrogen, NY) supplemented with penicillin (100 U/ml), streptomycin (100 μg/ml) and 5 or 10% fetal bovine serum (FBS, Atlanta Biological, Lawrenceville, GA), referred to here as complete DMEM. The cultured cells were kept in 100mm tissue culture dishes at 37°C in a humidified atmosphere containing 5% CO_2_.

### Design of animal experiment

All animal protocols used in this study were reviewed and approved by the PNU-Institutional Animal Care and Use Committee (PNU-IACUC, approval number PNU-2018-1955). Male three C57Bl/6N stocks (6-weeks-old) were obtained from three different sources. The C57BL/6NKorl mice were kindly provided by the Department of Laboratory Animal Resources of the National Institute of Food and Drug Safety Evaluation (NIFDS, Chungju, Korea). The other two stocks of C57BL/6N mice (C57BL/6NA and C57BL/6NB) were purchased from vendors located in the United States (Vendor A) and Japan (Vendor B), respectively. All C57BL/6N mice were maintained and treated at the Animal Resource Center of Pusan National University, which is certified by the Food and Drug Administration (FDA) as accredited unit number 000231, and Association for Assessment and Accreditation of Laboratory Animal Care (AAALAC) International according to the National Institutes of Health guidelines (Accredited Unit Number; 001525). During the experiment, all mice were maintained in a specific pathogen-free (SPF) state under a strict light cycle (lights on at 08:00 h and off at 20:00 h), at 23 ± 2°C and 50 ± 10% relative humidity. Animals were provided with *ad libitum* access to a standard irradiated chow diet (Samtako Biokorea Inc., Osan, Kyungido).

Mice were randomly divided into five groups for one C57BL/6N stock and LLC Cells (indicated cell numbers) in 1x PBS (100 μL) were subcutaneously injected into the right flanks of mice. All mice were dissected after natural death. For the survival curve, the mouse reached natural death, and tumor volumes were measured once every 2 days, using the formula (width^2^ x length)/2. For examining the tumors, growth of the tumor was observed up to 30 days, measuring the volume every 2 days, after which the mice were euthanized and samples collected. To measure the effect of anticancer drugs, tumors were induced by subcutaneous injection of LLC1 cells (5 × 10^5^ cells) in C57BL/6NKorl, C57BL/6NA and C57BL/6NB mice, followed by administering three different dose (100 ug/kg (LCP), 1 mg/kg (MCP), 5 mg/kg (HCP)) of an anti-cancer drug (Cisplatin), thrice a week. Malignant tissues, were subsequently collected and analyzed. When mice showed signs of morbidity, defined by the animal study protocol (e.g. short of breathiness, difficulty in moving, Rapid weight loss of 15–20% within a few days), they reached their endpoint and were euthanized for further study.

### Histological analysis

Tissues were excised from tumor bearing mice, fixed in 10% formalin, embedded in paraffin wax, processed routinely, and sectioned into 4 μm thick slices. Sections were then stained with hematoxylin and eosin (H&E), and their histopathological features were examined by light microscopy (Leica Microsystems, Wetzlar, Germany).

Immunohistochemical analysis (IHC) for measuring cell proliferation was assessed in malignant tissues excised from experimental animals and fixed in formaldehyde for 48 h. Fixed cancer tissues were embedded in paraffin blocks after dehydration, sliced to a thickness of 4 μm using Leica Microtome (Leica Microsystems), and collected on slides. For immunohistochemistry, slides were subjected to xylene and ethanol dehydration, followed by 3% H_2_O_2_ for 10 min, washed with 1x PBS, and blocked using 10% BSA. The fixed sections were then probed with Ki-67 anti-body (Novus Biologics, Littleton, CO, USA) prepared in 1% BSA at a concentration of 1:10, incubated overnight at 4°C, and subsequently treated with a secondary antibody (Biotinylated goat anti-rabbit IgG) and HRP streptavidin (Histostain-Plus Kit, Zymed, San Francisco, CA, USA). Finally, the amount of Ki-67 peptide was observed using a BX50F-3 microscope (Olympus, Tokyo, Japan) after inducing a color reaction using stable 3,3′-diaminobenzidine (DAB, Invitrogen).

### Western blot

Total proteins prepared from the tumor tissue were separated by 4–20% sodium dodecyl sulfate-polyacrylamide gel electrophoresis (SDS-PAGE) for 2 h, after which the resolved proteins were transferred to nitrocellulose membranes for 2 h at 40 V. Each membrane was then incubated separately, overnight at 4°C, with the following primary antibodies: anti-p53 (Sigma-Aldrich Co.), anti-p27 (Sigma-Aldrich Co.), anti-bax (Abcam, Cambridge, UK), anti-Bcl2 (Abcam), caspase-3 (Cell Signaling Technology, Danvers, MA, USA), anti-MMP2 (Santa Cruz Biotechnology, Inc. Santa Cruz, CA, USA), anti-VEGF (Abcam), and anti-β-actin (Cell Signaling Technology). The membranes were then washed with washing buffer (137 mM NaCl, 2.7 mM KCl, 10 mM Na_2_HPO4, and 0.05% Tween 20) and incubated with HRP-conjugated goat anti-rabbit IgG (Invitrogen) and HRP-conjugated goat anti-mouse IgG (Invitrogen) at a 1:1,000 dilution, at room temperature for 1 h. Membrane blots were developed using Amersham ECL Select Western Blotting detection reagent (GE Healthcare Piscataway, NJ, USA).

### Quantitative real-time polymerase chain reaction (PCR) analysis for cytokine gene expression

Quantitative real-time PCR was performed to assess the relative quantities of mRNA for IL-6, IL-10, and IL-1β. Total RNA molecules were isolated from frozen tumor tissues using RNA Bee solution (Tet-Test Inc., Friendswood, TX, USA). After quantification of the RNA concentration, the complement DNA (cDNA) was synthesized using a mixture of oligo-dT primer (Invitrogen, Carlsbad, CA, USA), dNTP and reverse transcriptase (Superscript II, Thermo Fisher Scientific, Inc., Waltham, MA, USA). Q-PCR was then conducted using a cDNA template and 2× Power SYBR Green (TOYOBO Co., Osaka, Japan). The reaction cycle at which PCR products exceeded this fluorescence intensity threshold during the exponential phase of PCR amplification was considered as the threshold cycle (CT).

### Statistical analysis

Statistical analyses were performed with SPSS for Windows, release 10.10, standard version (SPSS, Inc., Chicago, IL, USA). One-way analysis of variance (ANOVA) followed by Tukey’s post hoc test for multiple comparisons was performed to identify significant differences between groups. All values are reported as the mean ± S.E.M, and a *P* value < 0.05 is considered as significant.

## Result

### LLC tumor transplantation response of three different C57BL/6N stock

**Fig. 1:**
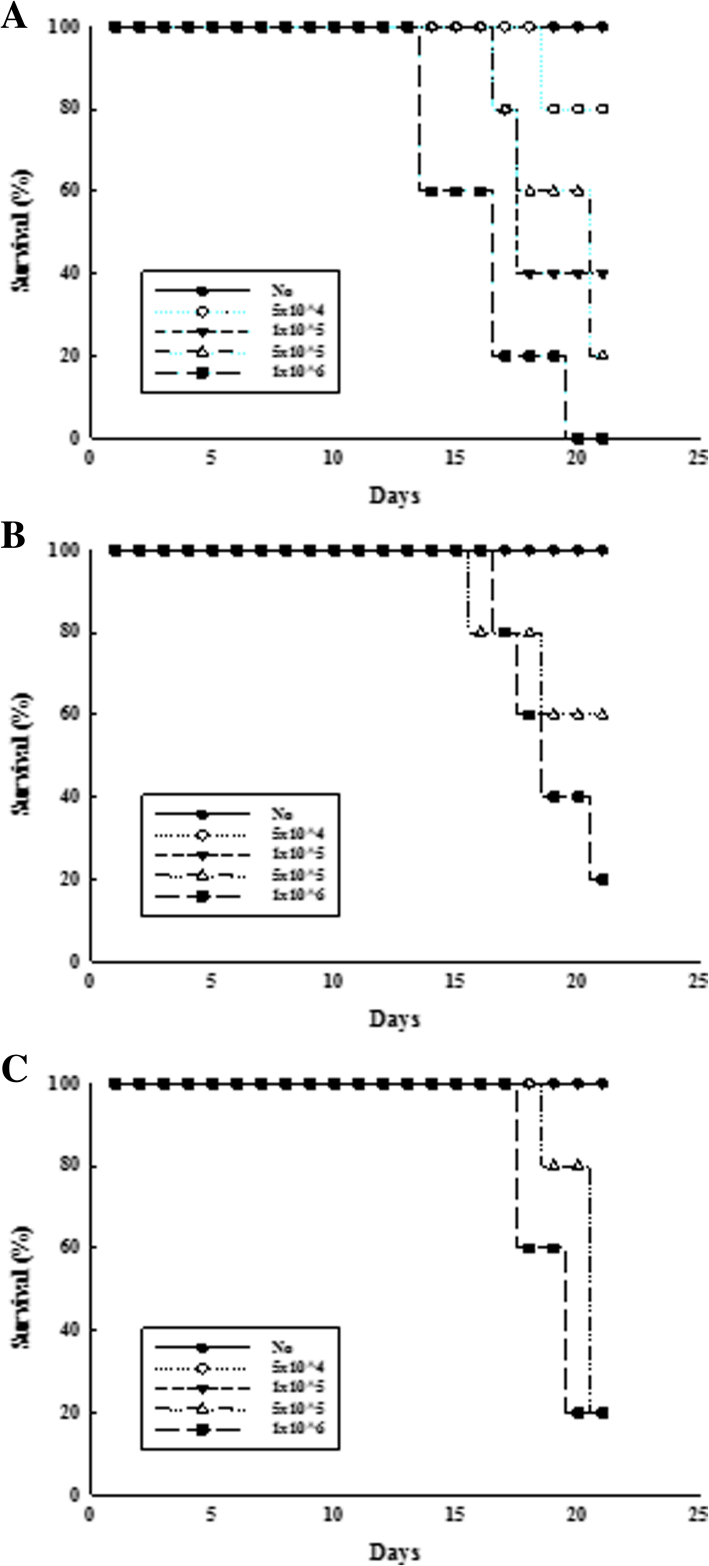
Comparison of the three C57BL/6N stocks on survival rate of mouse in LLC tumor cell transplant model. Four different numbers (5x10^4^, 1 x 10^5^, 5 x 10^5^ or 1 x 10^6^ cells) of LLC cells were subcutaneous injected in C57BL/6NKorl (**a**), C57BL/6NA (**b**), and C57BL/6NB (**c**), respectively. Each group consisted of 8 mice. The mouse survival was evaluated as described in Materials and Methods. Data represents the mean ± S.E.M of *n* = 8/group

**Fig. 2:**
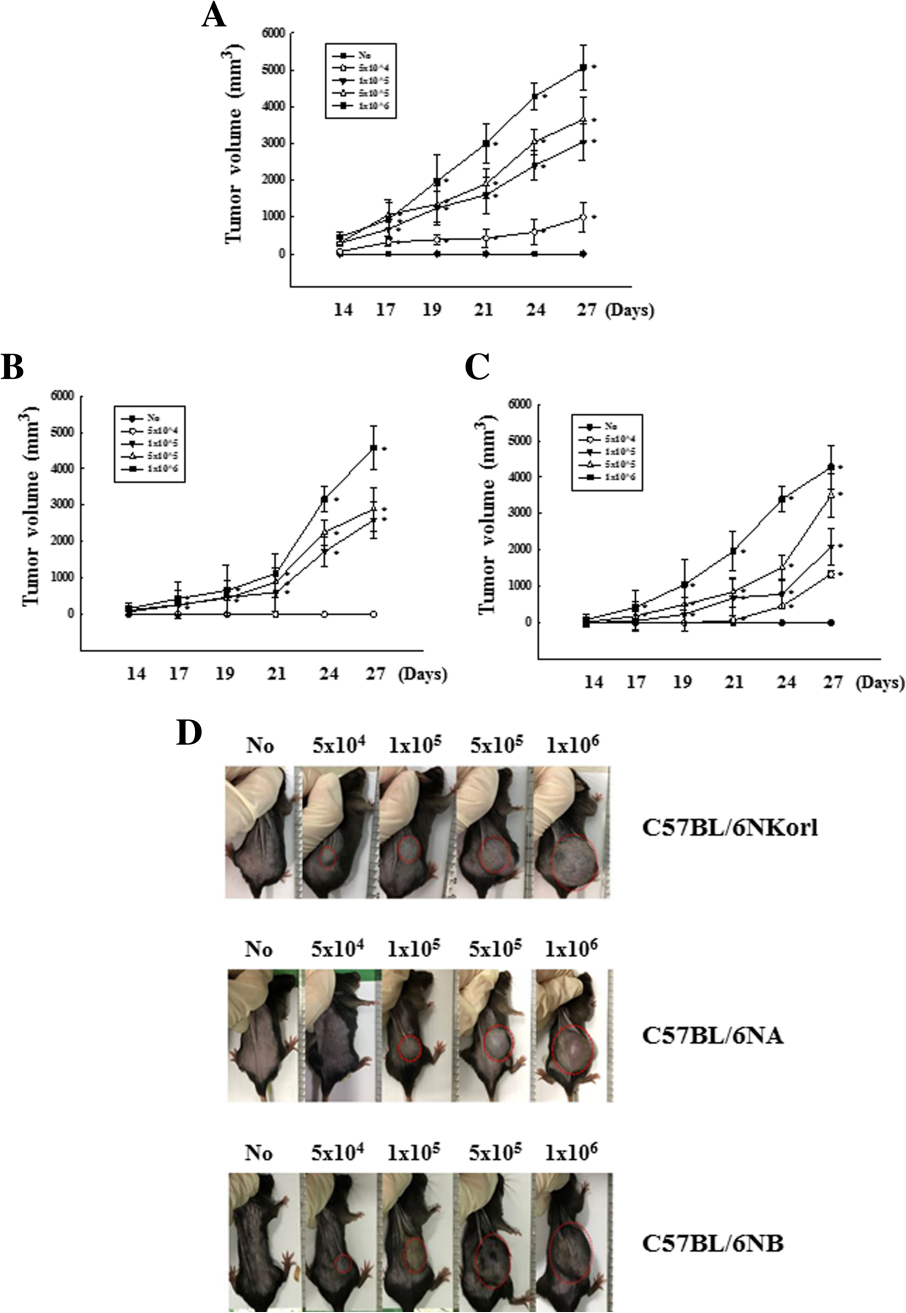
Comparison of the three C57BL/6 N stocks on tumor growth of mouse in LLC tumor cell transplant model. Four different numbers (5x10^4^, 1 x 10^5^, 5 x 10^5^ or 1 x 10^6^ cells) of LLC cells were subcutaneous injected in C57BL/6NKorl (**a**), C57BL/6NA (**b**), and C57BL/6NB (**c**), respectively. The tumor mass was evaluated at every 2 days. **d** Optic observation of growth tumors from each stock mice 27 days after LLC (indicated cells number/200 μl/body) transplantation. Each group consisted of 8 mice. The mouse growth was evaluated as described in Materials and Methods. Data represents the mean ± S.E.M of *n* = 8/group (*P < 0.05 versus No group)

In order to compare the responses of subcutaneously inoculated LLC tumors among the three C57BL/6N stocks, the mouse survival rate was employed to determine the response after inoculating four different concentrations of LLC cell numbers. Figure 1 shows survival curves of the C57BL/6N stocks after s.c. injection of LLC cells. As presented, the survival curves of C57BL/6NA and C57BL/6NB stocks are independent of the number of cells inoculated, but shows a similar tendency for mice mortality. However, the C57BL/6NKorl mouse shows a decrease in the survival rate relative to the number of cells inoculated. To evaluate the responsiveness of each C57BL/6N stock, we then assessed tumor growth and tumor volume with the number of cells inoculated. As shown in Fig. 2b and c, growth of tumors was over a prolonged duration in both C57BL/6NA and C57BL/6NB stocks (measurement beginning from 14 days), and increase in volume is not dependent on the number of cells inoculated. Contrarily, tumor growth in the C57BL/6Korl stock is relative to the number of cells inoculated, as assessed after 14 days (Fig. 2a). Visual observations of tumor volume show a similar pattern to that of the growth curve (Fig. 2d). In addition, results of measuring the weight of tumors obtained through dissection also reveal a tendency similar to the visual observation (Additional file 1: Figure S1). IHC was conducted using tumor tissues of the tumor bearing mice to observe cell proliferation as the cancer progresses. Results reveal that while the incidence of the cell proliferation factor (Ki-67) increases with the number of cancer cells, no differences were detected between the C57BL/6NKorl, C57BL/6NA and C57BL/6NB stocks (Additional file 2: Figure S2). These results suggest that C57BL/6NKorl shows better sensitivity in response to tumors than other stocks.

### Anti-cancer drug response of three different LLC-bearing C57BL/6N stocks

**Fig. 3:**
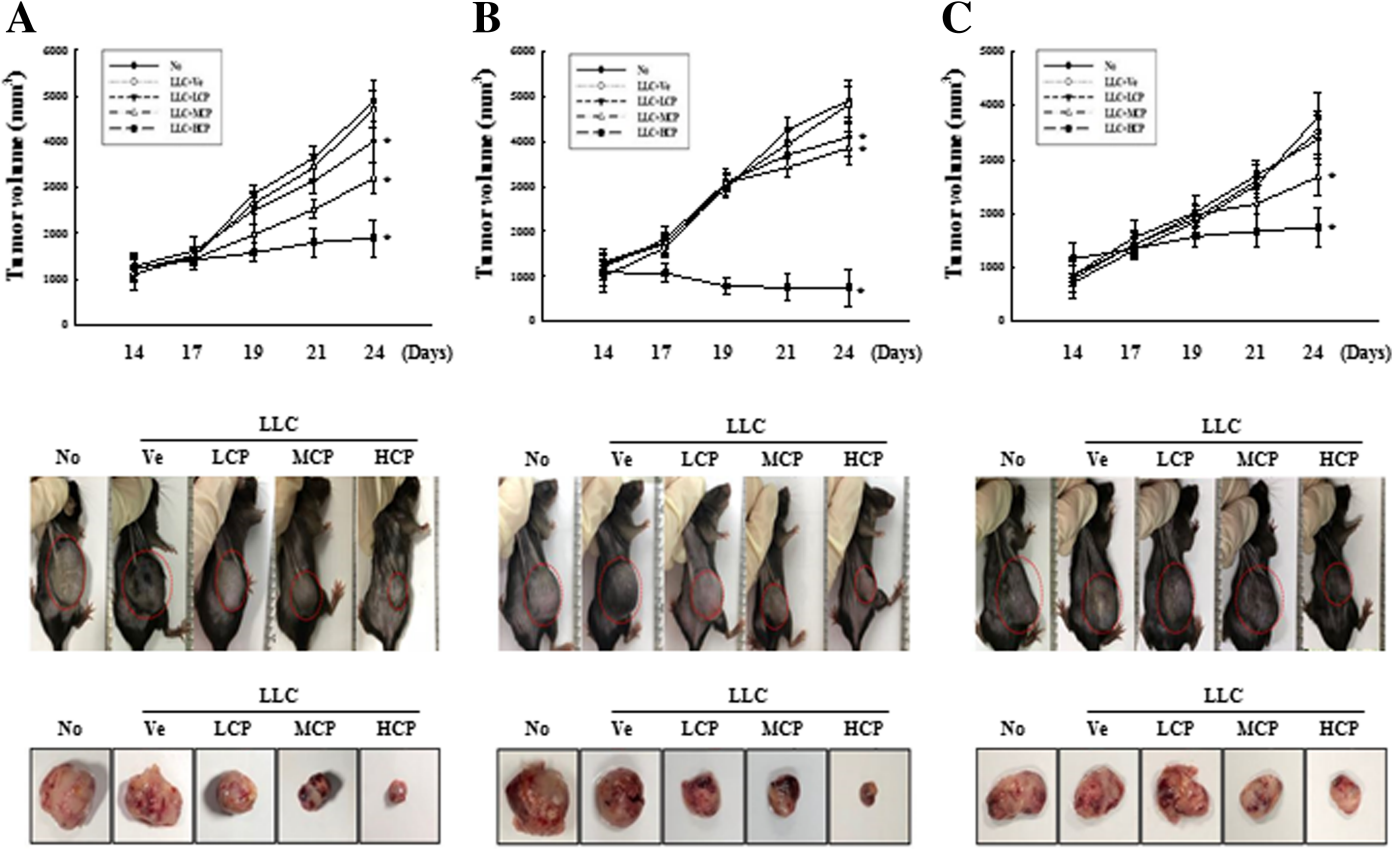
Comparison of cisplatin efficacy in inoculated LLC tumor cells among the C57BL/6NKorl, C57BL/6NA and C57BL/6NB. Three different concentration (100 μg/kg (LCP), 1 mg/kg (MCP) or 5 mg/kg (HCP)) of cisplatin were intra peritoneal injected in LLC cell bearing mice, C57BL/6NKorl (**a**), C57BL/6NA (**b**), and C57BL/6NB (**c**). The tumor mass was evaluated at every 2 days. Middle paragraph presents optic observation of growth tumors volume from each stock mice at 24 days after LLC transplantation. Bottom paragraph represents optic observation of isolated tumors mass. Isolation of tumor mass from each stock mice at 24 days after LLC transplantation. Each group consisted of 8 mice. Data represents the mean ± S.E.M of n = 8/group (*P < 0.05 versus LLC+Ve group)

Cisplatin (cis-diamine dichloroplatinum II) is a platinum-based anti-cancer drug widely used for the treatment of many solid tumors [16]. To evaluate the differences in the anti-cancer reactivity between the injected LLC cells and mouse stocks in a syngeneic mouse model using C57BL/6N mice, the responsiveness to the anti-cancer drug was investigated using cisplatin. From 14 days after administering cisplatin at concentrations of 100 μg/kg, 1 mg/kg and 5 mg/kg to the LLC-inoculated mice stocks, the tumor volume was visually observed and physically measured. We determined a decrease in the volume of cancer cells after administering cisplatin (Fig. 3). Furthermore, C57BL/6NKorl had a better response to decreased cancer volume after exposure to the anti-cancer drug as compared to C57BL/6NA and C57BL/6NB. The size of the tumor obtained after dissection conducted at day 30 after drug administration revealed a dose-dependent decrease, similar to the results of volumetric and visual observations (Fig. 3). As presented in Additional file 3: Figure S3, the IHC results to determine Ki-67 expression (indicating cell proliferation) showed decreased expression levels with increasing concentration of the anti-cancer drug; however, no differences were observed between the C57BL/6NKorl and the C57BL/6NB groups.

### Anti-cancer drug molecular mechanism of three different LLC-bearing C57BL/6N stocks

**Fig. 4:**
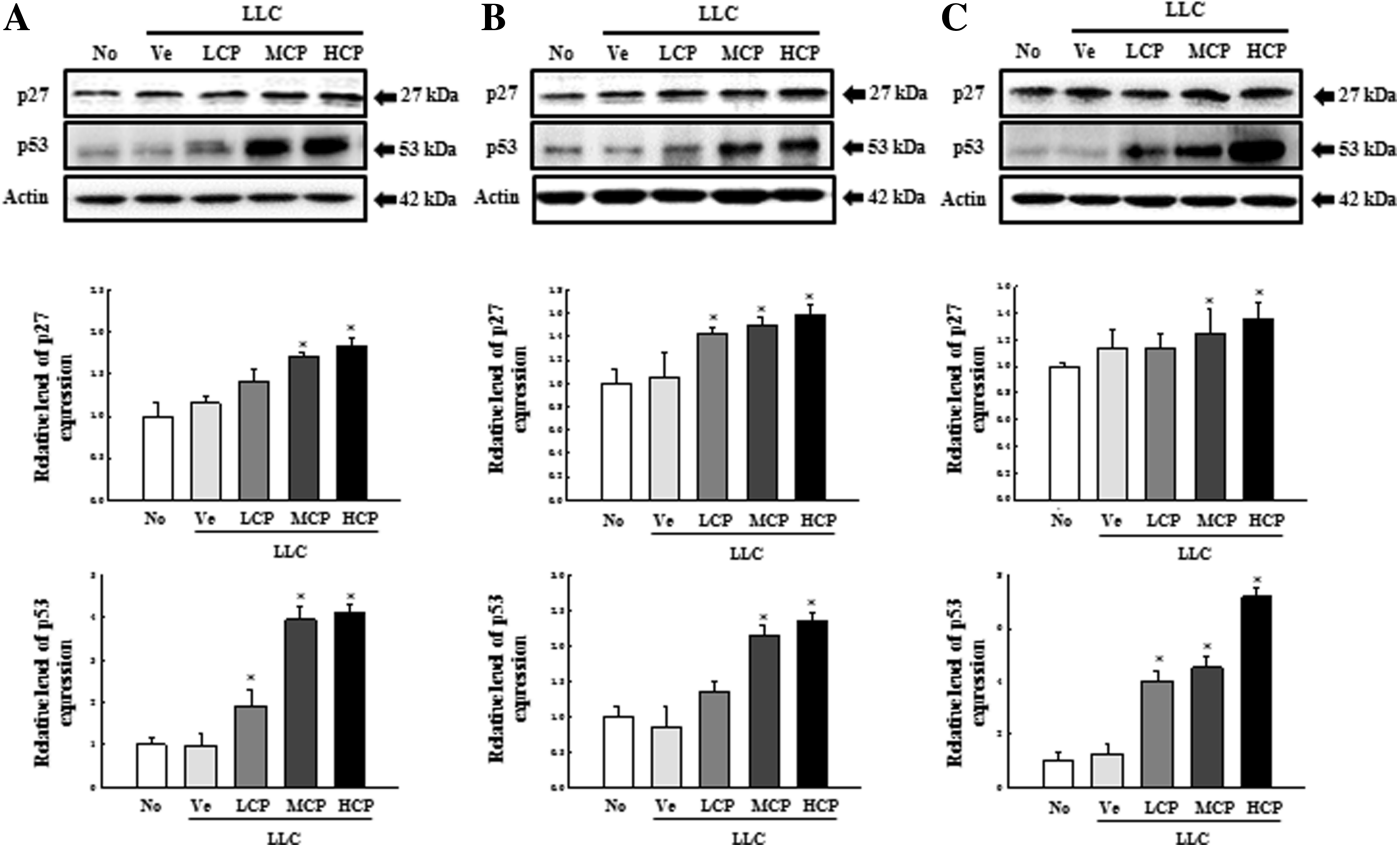
Comparison of p27 and p53 protein expression by cisplatin in tumor samples from the LLC cell bearing C57BL/6N mice stocks. Relative protein expression levels were measured using the western blotting. **a**, **b**, and **c** plots present the relative protein expression level of tumor tissue from cisplatin-treated tumor bearing C57BL/6NKorl (**a**), C57BL/6NA (**b**), and C57BL/6NB (**c**) stocks, respectively. Data represents the mean ± S.E.M of *n* = 8/group (*P < 0.05 versus LLC+Ve group)

**Fig. 5:**
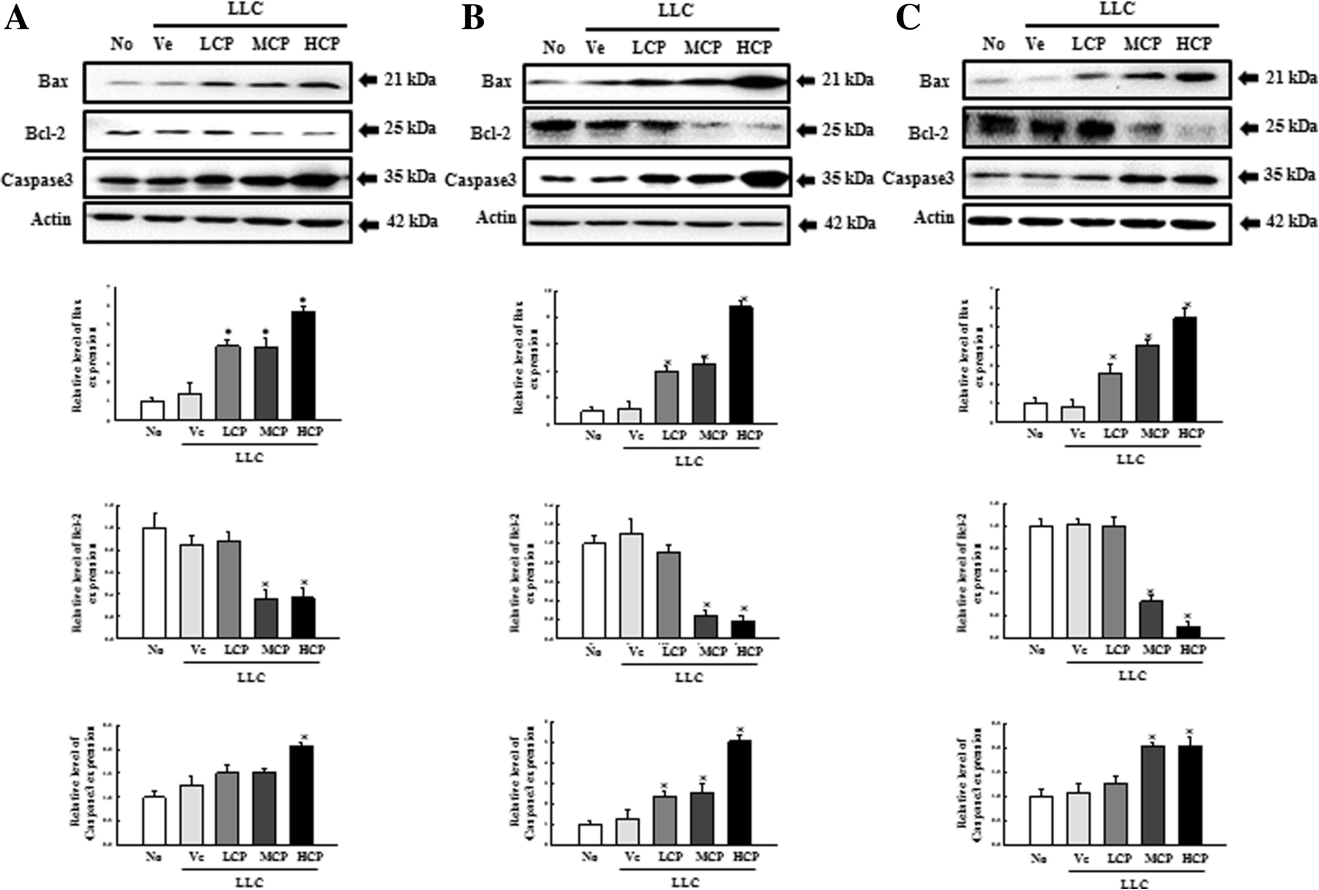
Determination of cisplatin-induced apoptosis in tumor tissue collected from LLC cell bearing C57BL/6N mice stocks using comparing the Bax/Bcl-2 protein ratio. Total lysates were prepared from the tumor tissues of cisplatin-treated tumor bearing C57BL/6NKorl (**a**), C57BL/6NA (**b**), and C57BL/6NB (**c**) mice of each group, as described in Materials and Methods. A total of 50 μg of protein per sample was immunoblotted with antibodies for each protein. Three samples were assayed in triplicate by western blotting. Data represents the means ± S.E.M of three replicates. *P < 0.05 versus LLC+Ve group

It is well known that administration of anti-cancer drugs induces DNA damage and destroys cancer cells by activating apoptosis in fast-growing cancer cells. In particular, increasing the expression of tumor suppressor proteins p27 and p53 following the administration of anti-cancer drugs is one of the key factors that results in cancer cell death. Therefore, the reactivity to cisplatin of C57BL/6N mice inoculated with LLC cells was compared by determining changes in the levels of p27 and p53. As shown in Fig. 4, administration of cisplatin increases the expression of p27 and p53 in a dose-dependent manner in all C57BL/6N stocks inoculated with LLC cells, with no significant difference observed among the three C57BL/6N stocks. Next, we examined for alterations in the expression of apoptosis-related proteins Bax, Bcl-2 and caspase3 after exposure of the anti-cancer drug to LLC-administered mice. The treatment of cisplatin in LLC-inoculated C57BL/6N mice showed increased expression of Bax and caspase3 (both inducers of apoptosis) in a dose-dependent manner. The expression of Bcl-2, an anti-apoptosis protein, was correspondingly reduced in a dose-dependent manner (Fig. 5). However, there were no significant differences among the three C57BL/6N stocks.

Cancer metastasis is the main reason resulting in a serious prognosis for cancer patients. Hence, administering anti-cancer drugs that inhibit metastasis is also critical. We, therefore, examined the effects of the anti-cancer drug on the expression of MMP2 and VEGF, a factor associated with cancer metastasis, in LLC-inoculated C57BL/6N mouse stocks. Additional file 4: Figure S4 shows that administration of cisplatin significantly reduces the expression of the metastasis related proteins in a dose-dependent manner, with no significant difference among the three C57BL/6N stocks. Next, to analyze the immunological changes resulting from administration of the anti-cancer drug in the C57BL/6N mouse stocks, we evaluated the expressions of IL-1β, IL-6 and IL-10 gene isolated from tumor tissue by real-time PCR. The results show a tendency to decrease as the concentration of the anti-cancer drug increases, with very similar reactions observed in all three stocks of mice (Additional file 5: Figure S5). Taken together, these results indicate that the effects of chemotherapy treatment are the same in the C57BL/6NKorl stock when compared to the two commercially available C57BL/6N stocks.

## Discussion

In this study, we undertook to identify differences between C57BL/6N mouse stocks among the commonly used mouse syngeneic models. We believe that this study will help to understand the differences within C57BL/6N stocks by comparing the tumor growth patterns and response to anti-cancer drugs, thereby facilitating in choosing the appropriate mouse model. The syngeneic mouse model has the following limitations: 1) the use of established tumors rather than spontaneous generation, 2) differences in tumor microenvironment, and 3) rapid growth of transplanted tumors [17]. Thus, limitations applicable to clinical tumor research are clear, as compared to the xenogeneic tumor models or genetically engineered mouse models. Despite these limitations, the syngeneic mouse model has been useful in recent primary researches on systemic and immunological studies due to its inherent advantages, such as the reduction of the experimental period due to rapid growth of tumor cells and low cost of research.

Cisplatin is an effective anti-cancer drug discovered in 1965, proven to have anti-cancer activity in mouse models and used in the treatment of solid tumors such as testicular cancer, ovarian cancer, cervical cancer and parietal cancer since its approval by the Food and Drug Administration in 1978 [18–20]. It includes primary treatment of solid tumors, as well as secondary therapy after surgery or radiation treatment. In addition, similar substances such as carboplatin and oxaliplatin, are currently being used. Adverse events associated with cisplatin include dose-limiting toxicity, nephrotoxicity, and drug resistance; many studies have also focused on cisplatin’s drug-target interaction, cell pharmacology, and drug dynamics. Research is underway to minimize the toxicity and bypass immunity by developing a similar skeleton as cisplatin for marginal ablation. The anti-tumor mechanism involves the interaction with chromosomal DNA to induce DNA damage, eventually leading to tumor cell death [21–27]. This study, therefore, used the representative anti-cancer drug cisplatin, which is known to react well to individual C57BL/6N stock in a syngeneic mouse model using LLC cells. Our study focused on investigating the molecular mechanisms in which cisplatin functions. We first examined the expression of tumor suppressor genes p27 and p53 (Fig. 4). p53 is phosphorylated and activated by DNA damage [28, 29]. p53 activates genes related to cell cycle progression, DNA recovery, and cell apoptosis. Several studies have shown that cisplatin activates wild type p53 in tumors [30–32]. p27 and various cell cycle related gene are involved in the p53-mediated apoptosis [33]. Thus, as shown in Figs. 4 and 5, changes in the expression of p53 and p27 as well as in apoptosis-related genes by cisplatin treatment in the syngeneic tumor model of C57BL/6N mouse was dependent on cisplatin concentration. This was an important factor in comparing the responsiveness of anti-cancer drugs.

It is well-known that the tumor microenvironment has an inflammatory mediator accumulation and mechanism for cell penetration. In particular, tumor sites feature chronic inflammatory conditions and there exists a significant correlation between chronic inflammation and tumor invasions, metastasis and chemotherapy [33]. As expected, changes in the manifestation of cytokines related to inflammation in the syngeneic model of C57BL/6N stocks by cisplatin showed reduction in the concentration of cytokines in a dose-dependent manner (Additional file 5: Figure S5). However, there were no significant differences among the C57BL/6N stocks, such as changes in proteins related to tumor apoptosis and changes in the expression of tumor suppressor genes as shown in Figs. 4 and 5, indicating that the C57BL/6NKorl stock can replace the two commercially available stocks in researches to study the efficacy of anti-cancer drugs.

## Conclusion

Taken together, we evaluated the growth and survival of tumors in syngeneic mouse models of C57BL/6N stocks, established conditions for building a syngeneic mouse model in the C57BL/6N stocks by evaluating the growth and survival of the tumor, administered cisplatin to each C57BL/6N stock model to establish the responsiveness to cancer drugs, such as tumor growth and molecular biological aspect including expression of p27 and p57, apoptosis related genes, metastasis factors and inflammatory proteins, in tumor bearing C57BL/6N stocks. Taken together, our results indicate that for all three C57BL/6N stocks, this syngeneic model has similar functionality and reactivity to anti-cancer drugs. Our results suggest that the C57BL/6NKorl mouse and C57BL/6N mouse from other commercial suppliers can be widely applied to produce a syngeneic model through tumor transplants.

## Additional files

Additional file 1: **Figure S1.** Comparison of the three C57BL/6N stocks on tumor mass of mouse in LLC tumor cell transplant model. Four different numbers (5x10^4^, 1 x 10^5^, 5 x 10^5^ or 1 x 10^6^ cells) of LLC cells were subcutaneous injected in C57BL/6NKorl (a), C57BL/6NA (c), and C57BL/6NB (c). Bottom paragraph represents optic observation of isolated tumors mass. Isolation of tumor mass from each stock mice at 27 days after LLC (indicated cells number/200 μl/body) transplantation. Each group consisted of 8 mice. The mouse growth was evaluated as described in Materials and Methods. Data represents the mean ± S.E.M of *n* = 8/group (*P < 0.05 versus no group). (TIF 106 kb)

Additional file 2: **Figure S2.** Comparison of the three C57BL/6N stocks on tumor cell proliferation of mouse in LLC tumor cell transplant model using Ki-67 staining. Four different numbers (5x10^4^, 1 x 10^5^, 5 x 10^5^ or 1 x 10^6^ cells) of LLC cells were subcutaneous injected in C57BL/6NKorl, C57BL/6NA, and C57BL/6NB, respectively. The graph presents the relative expression level of Ki-67 on tumor tissue from C57BL/6NKorl, C57BL/6NA and C57BL/6NB stocks, respectively. Each group consisted of 8 mice. Data represents the mean ± S.E.M of *n* = 8/group (*P < 0.05 versus No group). (TIF 300dpi)

Additional file 3: **Figure S3.** Comparison of the three C57BL/6N stock for tumor cell proliferation in mice with anti-cancer drug administration in a LLC tumor cell transplant model using ki-67 staining. LLC cell 5x10 5 was treated with indicated concentrations of cisplatin after subcutaneous injections in C57BL/6NKorl, C57BL/6NA, and C57BL/6NB. (d) The graph presents the relative expression level of Ki-67 on tumor tissue from C57BL/6NKorl, C57BL/6NA and C57BL/6NB stocks, respectively. Each group consisted of 8 mice. Data represents the mean ± S.E.M of n = 8/group (*P < 0.05 versus no group). (TIF 755 kb)

Additional file 4: **Figure S4.** Comparison of metastasis-related proteins expression in tumor tissue collected from LLC cell bearing C57BL/6N mice stocks. Western blotting represent relative level of MMP2 and VEGF protein in the tumor tissue from each C57BL/6N stock. The graph display the relative level of protein expression in tumor tissues from cisplatin-treated LLC cells bearing C57BL/6NKorl, C57BL/6NA, and C57BL/6NB stocks, respectively. *P < 0.05 versus LLC+Ve group (TIF 300dpi)

Additional file 5: **Figure S5.** Differing inflammatory responses of cisplatin in tumor tissue collected from LLC cell bearing C57BL/6N mice stocks. The mRNA levels of inflammation related proteins (IL-1 β, IL-6 and IL-10) were measured by real-time PCR using specific primers. Each panel represents the mRNA expression level of inflammation related proteins among tumor bearing C57BL/6NKorl (a), C57BL/6NA (b), and C57BL/6NB (c) mice after treatment with cisplatin or vehicle (*P < 0.05 versus LLC+Ve group). (TIF 300dpi)

## Data Availability

Available.

## Abbreviations

LLC cells: Lewis lung carcinoma cells
Cisplatin: Cis-diamine dichloroplatinum (II), Pt (NH_3_)_2_Cl_2_
IHC: Immunohistochemical analysis
