# Supplementary figures and images for: Comparison of responsiveness to cancer development and anti-cancer drug in three different C57BL/6N stocks
Source: Lab Anim Res. 2019 Oct 4;35:17. doi: 10.1186/s42826-019-0015-z (PMC7081605; doi:10.1186/s42826-019-0015-z)

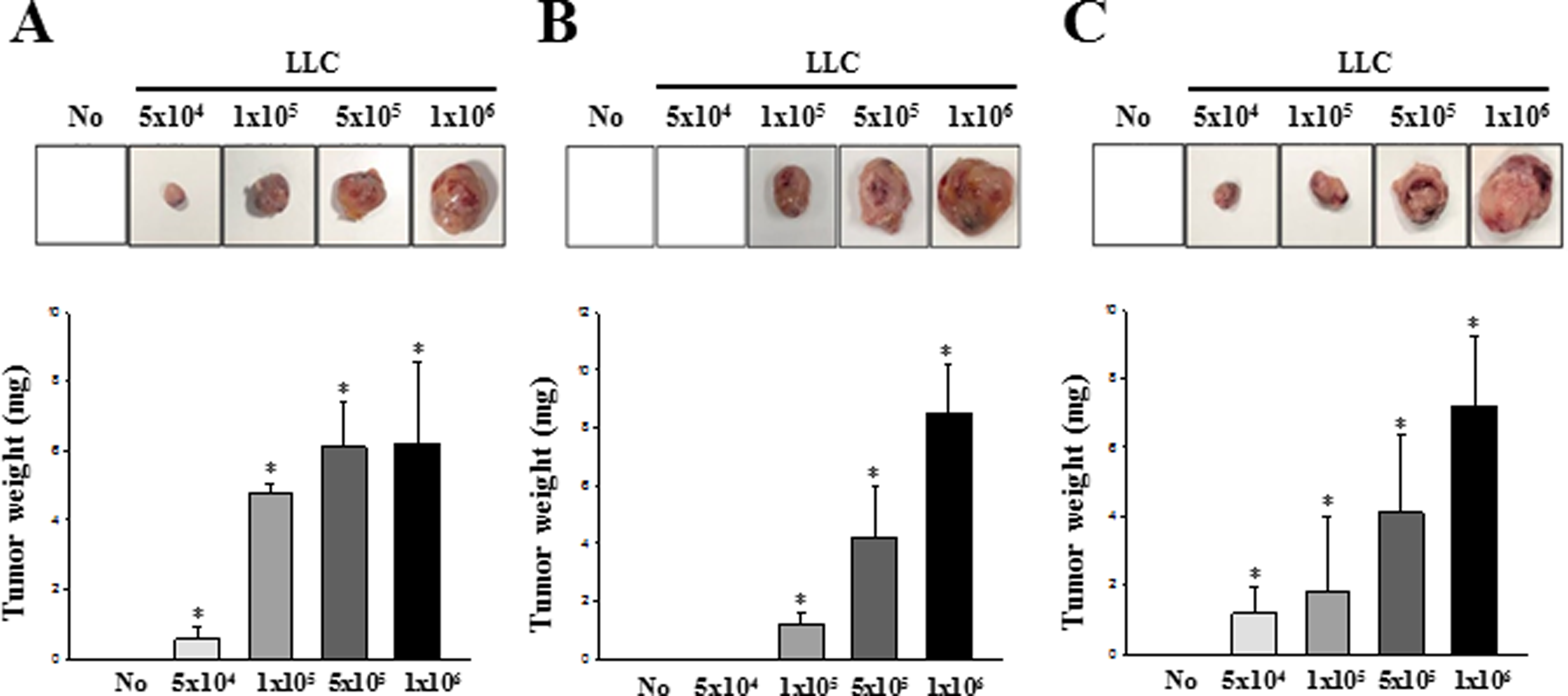

Supplement: Supplementary file 1 — Figure S1. Comparison of the three C57BL/6N stocks on tumor mass of mouse in LLC tumor cell transplant model. Four different numbers (5x10^4^, 1 x 10^5^, 5 x 10^5^ or 1 x 10^6^ cells) of LLC cells were subcutaneous injected in C57BL/6NKorl (a), C57BL/6NA (c), and C57BL/6NB (c). Bottom paragraph represents optic observation of isolated tumors mass. Isolation of tumor mass from each stock mice at 27 days after LLC (indicated cells number/200 μl/body) transplantation. Each group consisted of 8 mice. The mouse growth was evaluated as described in Materials and Methods. Data represents the mean ± S.E.M of n = 8/group (*P < 0.05 versus no group). (TIF 106 kb) [file 42826_2019_15_MOESM1_ESM.tif]

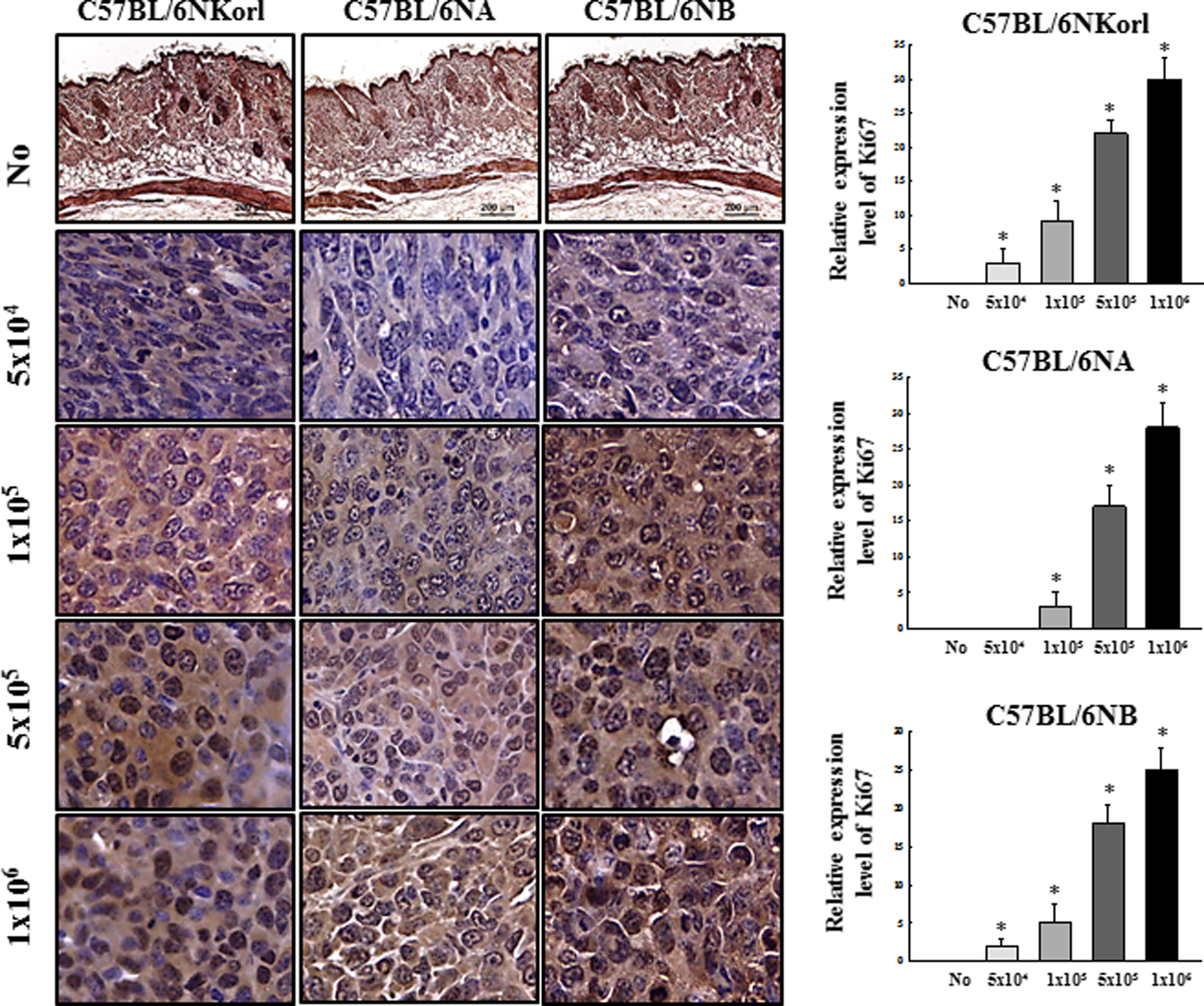

Supplement: Supplementary file 2 — Figure S2. Comparison of the three C57BL/6N stocks on tumor cell proliferation of mouse in LLC tumor cell transplant model using Ki-67 staining. Four different numbers (5x10^4^, 1 x 10^5^, 5 x 10^5^ or 1 x 10^6^ cells) of LLC cells were subcutaneous injected in C57BL/6NKorl, C57BL/6NA, and C57BL/6NB, respectively. The graph presents the relative expression level of Ki-67 on tumor tissue from C57BL/6NKorl, C57BL/6NA and C57BL/6NB stocks, respectively. Each group consisted of 8 mice. Data represents the mean ± S.E.M of n = 8/group (*P < 0.05 versus No group). (TIF 300dpi) [file 42826_2019_15_MOESM2_ESM.tif]

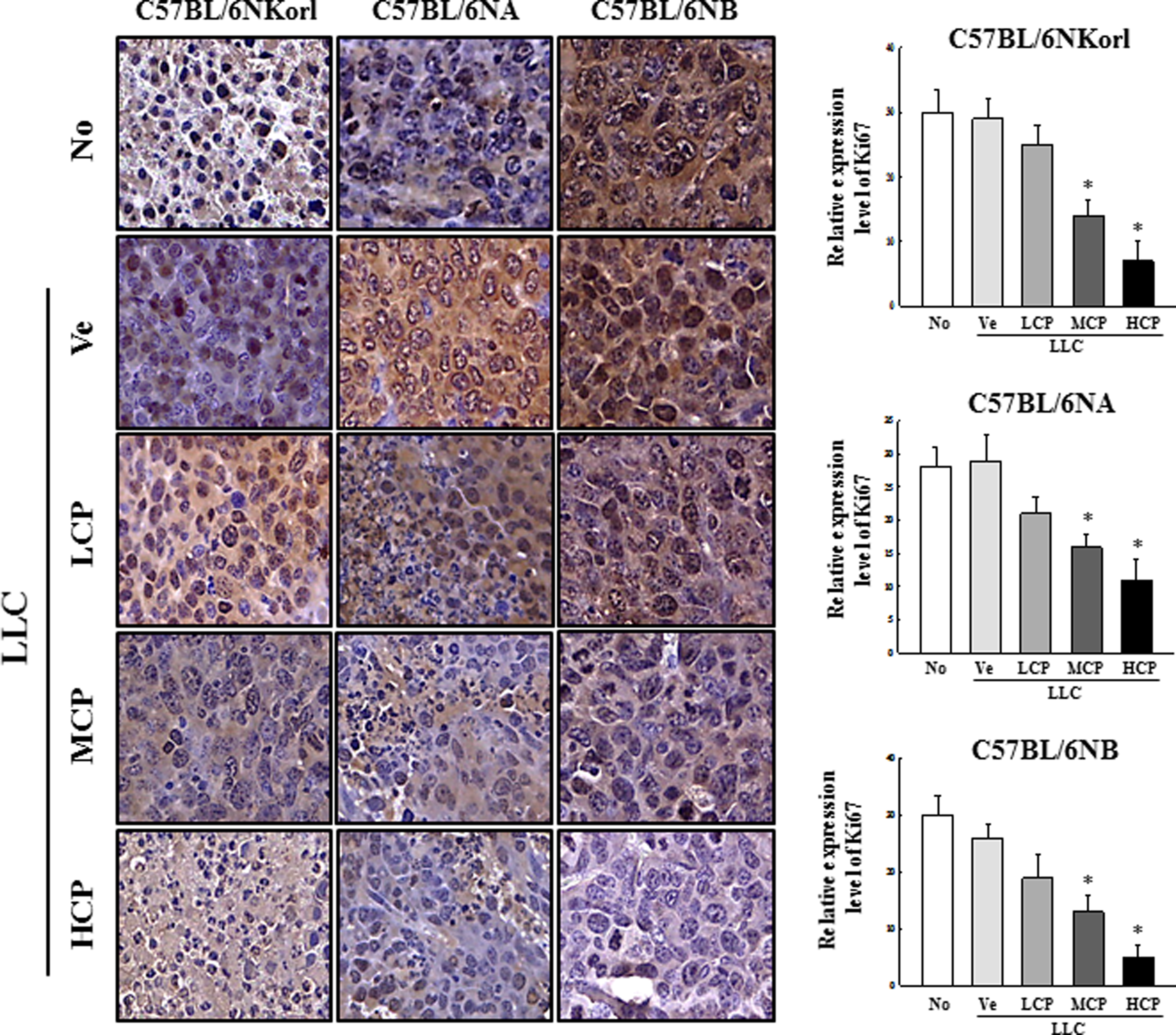

Supplement: Supplementary file 3 — Figure S3. Comparison of the three C57BL/6N stock for tumor cell proliferation in mice with anti-cancer drug administration in a LLC tumor cell transplant model using ki-67 staining. LLC cell 5x10 5 was treated with indicated concentrations of cisplatin after subcutaneous injections in C57BL/6NKorl, C57BL/6NA, and C57BL/6NB. (d) The graph presents the relative expression level of Ki-67 on tumor tissue from C57BL/6NKorl, C57BL/6NA and C57BL/6NB stocks, respectively. Each group consisted of 8 mice. Data represents the mean ± S.E.M of n = 8/group (*P < 0.05 versus no group). (TIF 755 kb) [file 42826_2019_15_MOESM3_ESM.tif]

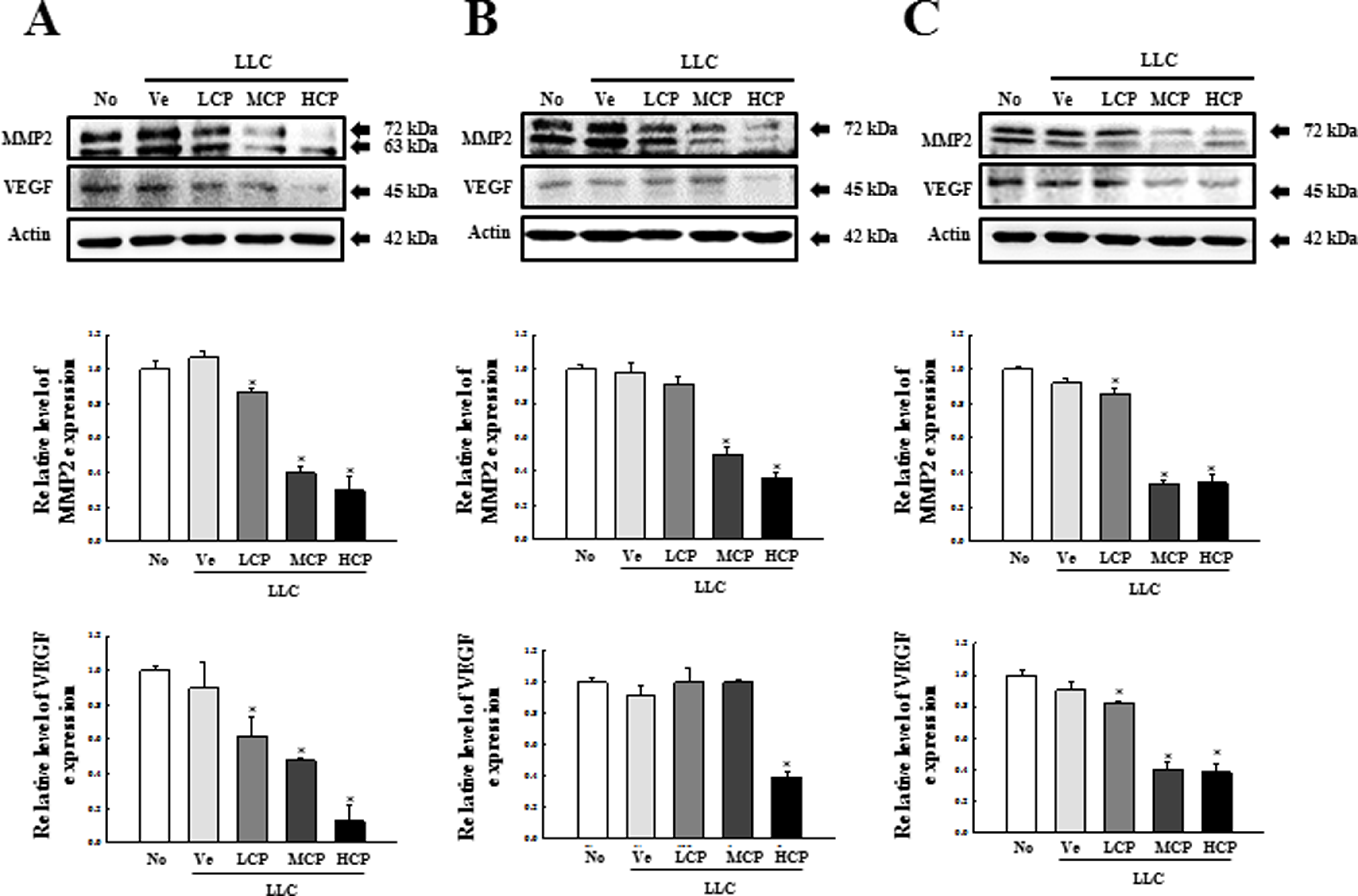

Supplement: Supplementary file 4 — Figure S4. Comparison of metastasis-related proteins expression in tumor tissue collected from LLC cell bearing C57BL/6N mice stocks. Western blotting represent relative level of MMP2 and VEGF protein in the tumor tissue from each C57BL/6N stock. The graph display the relative level of protein expression in tumor tissues from cisplatin-treated LLC cells bearing C57BL/6NKorl, C57BL/6NA, and C57BL/6NB stocks, respectively. *P < 0.05 versus LLC+Ve group (TIF 300dpi) [file 42826_2019_15_MOESM4_ESM.tif]

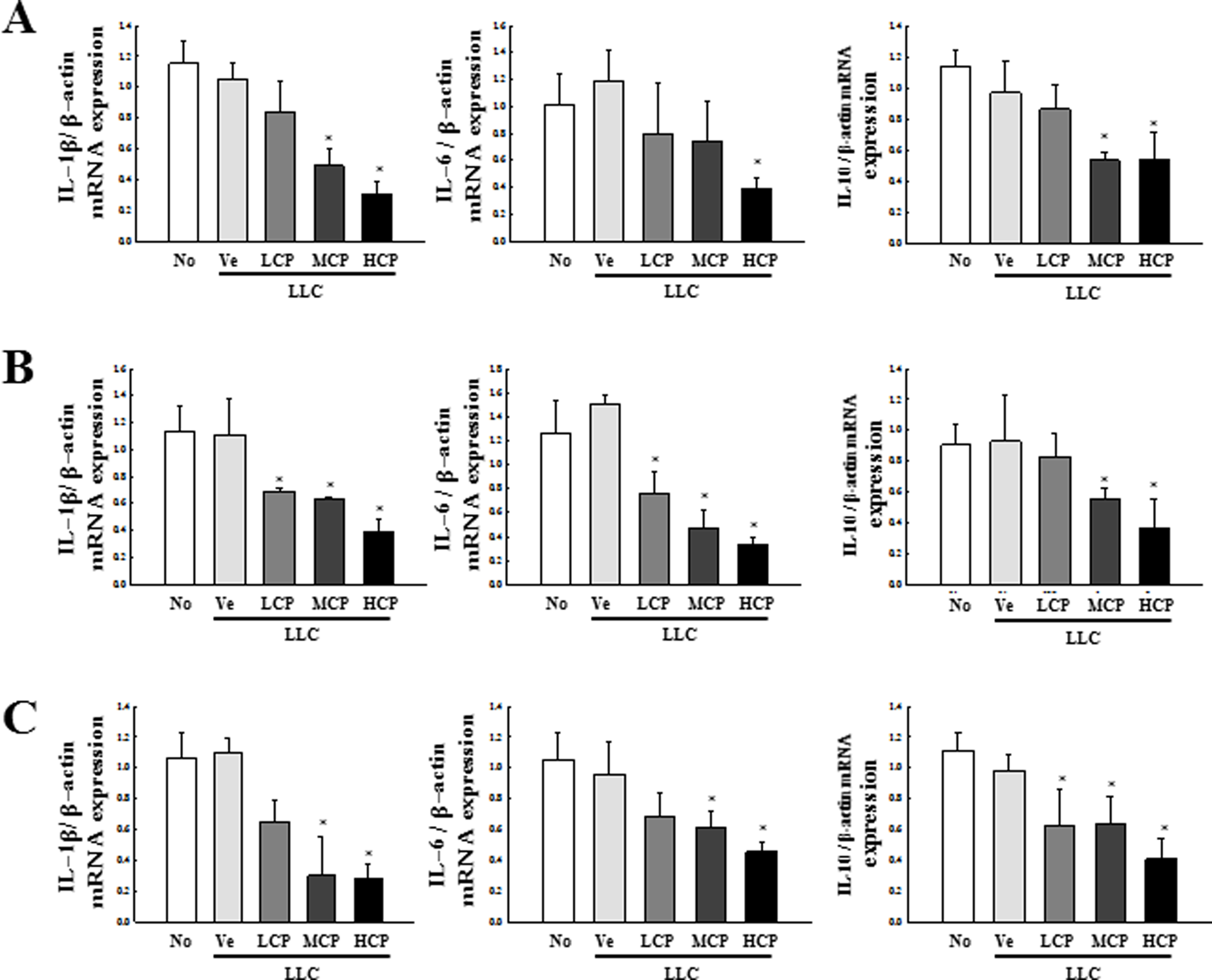

Supplement: Supplementary file 5 — Figure S5. Differing inflammatory responses of cisplatin in tumor tissue collected from LLC cell bearing C57BL/6N mice stocks. The mRNA levels of inflammation related proteins (IL-1 β, IL-6 and IL-10) were measured by real-time PCR using specific primers. Each panel represents the mRNA expression level of inflammation related proteins among tumor bearing C57BL/6NKorl (a), C57BL/6NA (b), and C57BL/6NB (c) mice after treatment with cisplatin or vehicle (*P < 0.05 versus LLC+Ve group). (TIF 300dpi) [file 42826_2019_15_MOESM5_ESM.tif]
